# Science by social media: Attitudes towards climate change are mediated by perceived social consensus

**DOI:** 10.3758/s13421-019-00948-y

**Published:** 2019-06-21

**Authors:** Stephan Lewandowsky, John Cook, Nicolas Fay, Gilles E. Gignac

**Affiliations:** 1 School of Psychological Science, University of Bristol, 12a Priory Road, Bristol, BS8 1TU UK; 2 University of Western Australia, Perth, Australia; 3 George Mason University, Fairfax, VA USA

**Keywords:** Social media, Science communication, Online disinformation, Perceived consensus

## Abstract

Internet blogs have become an important platform for the discussion of many scientific issues, including climate change. Blogs, and in particular the comment sections of blogs, also play a major role in the dissemination of contrarian positions that question mainstream climate science. The effect of this content on people’s attitudes is not fully understood. In particular, it is unknown how the interaction between the content of blog posts and blog comments affects readers’ attitudes. We report an experiment that orthogonally varied those two variables using blog posts and comments that either did, or did not, support the scientific consensus on climate change. We find that beliefs are partially shaped by readers’ perception of how widely an opinion expressed in a blog post appears to be shared by other readers. The perceived social consensus among readers, in turn, is determined by whether blog comments endorse or reject the contents of a post. When comments reject the content, perceived reader consensus is lower than when comments endorse the content. The results underscore the importance of perceived social consensus on opinion formation.

Americans are now as likely to resort to the Internet as their primary source of science information as they are to rely on television (Su, Akin, Brossard, Scheufele, & Xenos, 2015). Leading science blogs can attract up to 1.5 million visitors and several thousand comments every month (Batts, Anthis, & Smith, 2008). Unlike conventional media, Internet blogs by design provide a platform for dynamic many-to-one and many-to-many dialogues: visitors can comment on an article or video,^1^ the author of a post can reply to commenters, and commenters can interact with each other. This multi-layered dynamic has changed the nature of information transfer, with readers no longer just consuming content passively but also actively contributing to on-line content.

However, the opportunity for authentic multi-party debate also opens the door to misleading, offensive, or inappropriate content. This darker side of blogs is of particular concern in the scientific arena: in addition to providing a platform for scientific discussion, Internet blogs also constitute a staging ground for the denial of well-established scientific findings (Briones, Nan, Madden, & Waks, 2012; Kata, 2012; Lewandowsky, Oberauer, & Gignac, 2013; Lewandowsky et al., 2015; Zimmerman et al., 2005). To illustrate, Lewandowsky, Ballard, Oberauer, and Benestad (2016) reported a blind test involving expert statisticians, who were presented with contrarian interpretations of climate data that were disguised as economic variables. The experts found those interpretations, including samples from contrarian blogs, to be misleading and inappropriate for policy advice. Similarly, although the safety and efficacy of childhood vaccinations are not subject to scientific dispute (van der Linden, Clarke, & Maibach, 2015), up to 71% of content returned by Google in response to the search term “vaccination” is slanted *against* the scientific consensus (Kata, 2010). Much of that contrarian content arises from blog posts and blog comments (Briones et al., 2012; Kata, 2012).

Blog readers therefore operate in a challenging environment in which they must evaluate the credibility of content from multiple competing, and often unknown, sources (Walther & Jang, 2012). Are blog posts trustworthy? Do comments on blog posts provide corrections of errors or do they introduce further erroneous content? Can comments reveal how posts are being received by other readers? Many of these questions have thus far escaped research attention, even though the need to examine the role of blog comments is brought into sharp focus by the proliferation of “sock puppets”, which are fake online identities controlled by a small group of operatives that can create an illusion of support for, or opposition to, an opinion (Bu, Xia, & Wang, 2013; Lewandowsky, 2011). During the U.S. presidential election of 2016, research has identified a substantial portion of all pro-Trump traffic on Twitter to have resulted from automated accounts (“tweetbots”), with automated pro-Trump traffic being at least four times as prevalent as automated pro-Clinton traffic (Kollanyi, Howard, & Woolley, 2016).

This article examines how blog posts and blog comments interact to affect readers’ attitudes and beliefs concerning the scientifically well-established finding that greenhouse gas emissions are warming the Earth, a phenomenon known as climate change or, more formally, anthropogenic global warming (AGW). Although there is no notable scientific dissent from this mainstream position (Cook et al., 2013, 2016), a sizeable and highly vocal segment of the public denies those facts for political or ideological reasons (Lewandowsky, Gignac, & Oberauer, 2013). Blogs are an integral component of climate-contrarian activities (Lewandowsky et al. 2013, 2015). Here we focus on how blogs may affect readers’ attitudes by altering the perceived prevalence of an opinion among readers. Several lines of theorizing and a large body of empirical work suggest that perceived social consensus can be a powerful agent in shaping and changing people’s attitudes.

Inspired partly by work on collective behavior in non-human animals (e.g., Galef & Giraldeau, 2001), there has been much recent research interest in social learning in humans (e.g., Kendal et al., 2018). A key insight of this research has been that people are finely attuned to social cues in their environment. Even very subtle cues, such as a few people staring at the top of a building in a public place, can cause others to change their behavior, for example by following suit and looking up themselves (Gallup et al., 2012). Similarly, the number of viewers of a YouTube video can be interpreted as a signal of the prominence of an issue in the public’s mind (Spartz, Su, Griffin, Brossard, & Dunwoody, 2017). Given that YouTube views and “likes” can be purchased in bulk for very little money on the black market (NATO StratCom COE, 2018), those effects may give rise to concern.

However, copying others’ behavior is neither universal nor necessarily always adaptive: Conformity may be advisable when asocial learning (i.e., individual exploration and learning directly from the environment) is impossible or costly, or when people are uncertain about their own views (Kendal et al., 2018). Under those circumstances, people tend to copy the behavior or opinion of a majority, and this tendency increases with the size of the majority (Muthukrishna & Henrich, 2019). The fact that conformity increases with the size of the majority is adaptive and rational because larger groups of independent actors will provide more reliable information than small groups (Muthukrishna, Morgan, & Henrich, 2016). When the size of the majority becomes overwhelming, conformity effects are also known as “consensus effects.” Much prior work has focused on how a social consensus can affect opinions relating to stereotypes and discrimination. For example, if a participant receives (experimentally manipulated) information about the predominant attitudes among his or her peers—viz. how they view minority groups—then the person’s own attitude tends to shift in the direction of the purported consensus (Puhl, Schwartz, & Brownell, 2005; Stangor, Sechrist, & Jost, 2001; Zitek & Hebl, 2007). The effect is enhanced if the consensus involves members of one’s in-group, and it can be long-lasting and is detectable outside the context of the initial manipulation (Stangor et al., 2001).

In the context of scientific issues such as climate change or vaccinations, providing information about a consensus among experts has similarly been shown to nudge people’s attitudes towards the scientific mainstream (Lewandowsky, Gignac, & Vaughan, 2013; van der Linden, Leiserowitz, Feinberg, & Maibach, 2015; van der Linden et al., 2015). Perception of the scientific consensus has been identified as a “gateway belief”; that is, a crucial conceptual underpinning of numerous climate-related beliefs (Ding, Maibach, Zhao, Roser-Renouf, & Leiserowitz, 2011; McCright, Dunlap, & Xiao, 2013; Stenhouse et al., 2014; van der Linden et al., 2015). Conversely, highlighting of only a few dissenting views on climate change can undermine the perception of a scientific consensus and the need for expert guidance during policy development, even when numeric information about the extent of expert agreement is available (Koehler, 2016).

It is largely unknown whether anonymous blog comments provide effective social consensus information. We are aware of only two studies that examined this issue (Stavrositu & Kim, 2015; Winter & Krämer, 2016), both of which considered the effect of contrarian comments on readers’ responses to a scientific post. In both cases, comments that opposed the post decreased the impact of the scientific content by altering the perceived social norm among readers. Specifically, (Stavrositu & Kim, 2015) presented participants with a blog post relating to skin cancer, and found that behavioral intentions (e.g., to get screened for skin cancer) were a function of perceived consensual intentions among “other readers”, which in turn were (weakly) determined by the type of blog comments: When comments supported the tenor of the post—i.e., medical information about skin cancer—participants perceived this as indicative of a consensus among readers and reported their own intentions accordingly. However, when comments were dismissive of the content, the perception of a consensus and behavioral intentions were reduced. Along similar lines, Winter and Krämer (2016) presented participants with a blog post that summarized the evidence for the adverse psychological effects of violent video games. The presence of dissenting comments was found to undermine support for the evidence presented in the post among participants who were not vested in the topic.

Taken together, the two studies point to the potentially corrosive effect of dissenting comments on people’s perceived reader-consensus and their own attitudes towards the scientific content. However, neither study manipulated the content of the blog post that preceded the comments, leaving open the question of how comments and post content interact. In particular, it remains unknown whether the effect of comments can be symmetrical: Can comments that dissent from a *contrarian* post, thereby endorsing the scientific mainstream position, enhance acceptance of the science in the same way that dissenting comments after a mainstream post might undermine support for the scientific position? Accordingly, our study fully crossed the type of post (endorsing or rejecting the scientific mainstream position) with the type of comment (endorsing or rejecting the mainstream). If blog comments shift opinions on post content by creating a social norm, as suggested by the results of Stavrositu and Kim (2015) and Winter and Krämer (2016), then they should exert a symmetrical effect irrespective of the content of the post.

We adopted this expectation as our working hypothesis in an experiment that orthogonally combined two types of blog post (endorsing and rejecting mainstream climate science) with two types of blog comments (endorsing and rejecting mainstream climate science). A representative sample of the American public participated in the experiment online and responded to questions about the blog post as well as climate change more generally after they had read both a post and an accompanying set of comments.

## Methods

### Design

The experiment featured a 2 × 2 between-participants design formed by orthogonally combining the type of blogpost (endorsing or rejecting AGW) and the nature of the accompanying comments (endorsing or rejecting AGW). Table 2 lists the test items that were used to form the dependent variables.

### Stimuli

Content was created by the team at the Skeptical Science blog (www.skepticalscience.com). Two blogposts were written, one endorsing AGW and the other rejecting AGW. The post that endorsed AGW summarized the consensual scientific evidence in lay people’s terms, and the post that opposed AGW was a concatenation of known contrarian talking points. For each blogpost, two threads of ten comments (by ten unique fictitious identities; e.g., “Grand Poobah”) were created. One thread only featured comments endorsing AGW and the other thread only contained comments rejecting AGW. Because incivility is known to be rife in the blogosphere and is also known to affect people’s attitudes in various ways (Anderson, Brossard, Scheufele, Xenos, & Ladwig, 2013; Borah, 2012; Ng & Detenber, 2005; Sobieraj & Berry, 2011), we controlled for those effects by ensuring that all posts and comments conformed to standards of civility. The two posts and their two streams instantiated the four conditions within the 2 × 2 design. The full text of the posts and comment streams are available at https://github.com/StephanLewandowsky/Blog-comments. Table 1 summarizes readability statistics for the two posts and the comment streams.

**Table 1:** Summary statistics for the stimuli used in the experiment

Post				Comments			
Type of post (TP)	Wordcount	F-K grade^a^	Flesch ease^b^	Type of comments (TC)	Wordcount	F-K grade^a^	Flesch ease^b^
Reject AGW	888	9.4	53.8	Endorse AGW	989	6.6	70.1
				Reject AGW	991	6.3	74.2
Endorse AGW	888	9.5	53	Endorse AGW	999	6.6	69.8
				Reject AGW	990	6.4	69.7

^a^ F-K grade = Flesch–Kincaid readability index (Kincaid, Fishburne, Rogers, & Chissom, 1975)

^b^ Original Flesch readability index (Flesch, 1948)

In all conditions, the post was followed by three yes–no comprehension questions: “The blog post you just read suggested that the climate has been changing due to changes in the ocean” (correct for the post rejecting AGW, incorrect for the post endorsing AGW); “The blog post you just read suggested that Bono (U2 singer) should have no business in politics” (always incorrect); and “The blog post you just read stated that atmospheric CO2 levels have risen 40% since pre-industrial times” (always correct). These comprehension questions (two of which had to be answered correctly for a participant to be included in the analysis) were followed by the comment stream. To mimic the way in which people likely interact with comments in real-life situations, we did not constrain participants’ reading time on the comments.

The comment stream, in turn, was followed by various test items, which are shown in Table 2 in the order in which they were presented. (Test items were followed by questions about age and gender, not shown in the table). The items queried people’s belief in their susceptibility to blog comments, their perceived consensus of opinion about the post among other readers, and their attitudes toward climate change and the perceived scientific consensus. The five AGW items (*agw1–agw5*) and the scientific consensus (*SciCons*) were taken from Lewandowsky et al., (2013), and the remaining items were developed for this study.

**Table 2:** Test items used in all conditions

Short label^a^	Item	Response scale^b^
*Unaffected*	My opinion about a blog post is completely unaffected by	SD to SA
	the comments made on the article by others.	
*ReaderCons*	Out of every 100 readers of this post, how many do you	Slider
	think support the basic argument made in this blog post?	
*Support*	Overall, I support the basic argument made in this blog post.	SD to SA
*agw1*	I believe that the climate is always changing and what we	SD to SA
	are currently observing is just natural fluctuation. (R)	
*agw2*	I believe that most of the warming over the last 50 years is	SD to SA
	due to the increase in greenhouse gas concentrations.	
*agw3*	I believe that the burning of fossil fuels over the last 50 years	SD to SA
	has caused serious damage to the planet’s climate.	
*agw4*	Human CO_2_ emissions cause climate change.	SD to SA
*agw5*	Humans are too insignificant to have an appreciable impact	SD to SA
	on global temperature. (R)	
*SciCons*	On a scale from 0% to 100%, in your opinion, how many	Slider
	climate scientists agree that human activity is causing global warming?	

^a^ Labels are provided for items that are entered into the final analysis and are used in the figures

^b^ Slider = respondents used a slider with end points 0 and 100 to enter a number; SD to SA = 5-point scale from Strongly Disagree (1) to Strongly Agree (5), with a Neutral (3) response option. Items marked with “(R)” were reverse-scored

### Participants and procedure

The study was conducted by Qualtrics.com (Provo, Utah), a firm that specializes in representative Internet surveys, in 2014 using a contractually agreed sampling plan. The contract requested a sample of 400 representative U.S. residents. Participants were members of a completely bipartisan panel of more than 5.5 million U.S. residents (as of January 2013), who were invited via propensity weighting to ensure approximate conformance to the U.S. Census distribution for age, gender, and region. Participants were randomly assigned to the four conditions (minimum *N* = 91, maximum *N* = 102). The overall sample size met the minimum sample size of 200 recommended for latent variable modeling estimated via maximum-likelihood (Boomsma and Hoogland, 2001), as well as the sample size of approximately 400 for expected moderately sized direct and indirect effects estimated within a mediation analysis (Wolf, Harrington, Clark, & Miller, 2013).

Only participants who completed all survey items and who passed at least two out of three comprehension questions after reading the blog post were included in the sample. Participants were compensated by Qualtrics with cash-equivalent points.

After providing informed consent, participants read the blogpost and comment stream as appropriate for the condition to which they were assigned and then responded to all test items. Participants who were exposed to the posts rejecting AGW received an additional debriefing after completing the test items that corrected the erroneous information that had been imparted in the blog post. Debriefing in those conditions was in turn followed by a few further test items that formed part of a different experiment and are not reported here.

## Results

### Data preprocessing

The data set delivered by Qualtrics contained completed records from 403 participants, ten of whom did not meet the criterion of passing at least two out of three comprehension questions. Those records were eliminated, yielding a final sample of 393 participants for analysis (198 male, 195 female). The mean age of the sample was 46 (median = 45, Q1 = 29, Q3 = 60).^2^ The data set is available at https://github.com/StephanLewandowsky/Blog-comments.

We next considered the time participants spent on reading the comments. Reading time for the comments varied considerably across participants, from 2.5 s to 4530 s (1 h 15 min). Jackson and McClelland (1979) reported adult reading speed estimates for college-level text that ranged from 33 words per minute (wpm) to 454 wpm. Those estimates translate into expected reading times ranging from around 130 s to 1800 s for the roughly 1000 words in our comment stream. Given that our comments were written in a colloquial style that would likely be easier to process than the texts used by Jackson and McClelland (1979), we decided to define “careful readers” as anyone who spent between 100 s and 1800 s processing the comments. All further analyses were conducted both on the full set of participants who passed the comprehension questions (*N* = 393) and a subset of careful readers (*N* = 183). With two exceptions noted below, the two analyses yielded qualitatively identical results except that all effects were more pronounced with the subset of careful readers. We therefore only report the analyses of the full sample.

### Tests of experimental effects

**Fig. 1:**
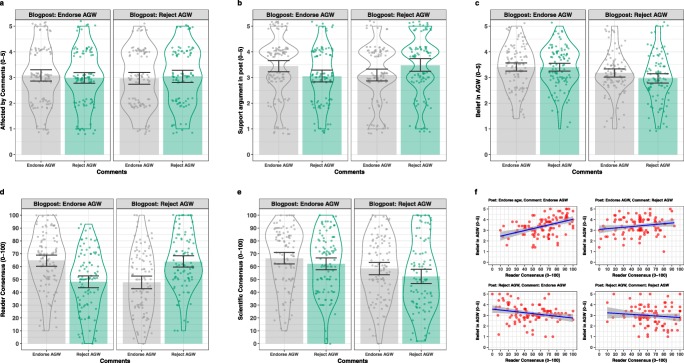
Summary statistics and distributional information for all dependent variables (see Table 2) across the four cells of the experimental design. *Bars* represent cell means and error bars are 95% bootstrapped (*N*= 1,000 samples) confidence intervals. Data points within violin plots are jittered to avoid over-printing. **a***Unaffected*; **b***Support*; **c***AGW*, which is average of responses to items *agw1, agw2, agw3, agw4*, and *agw5* after reverse scoring; **d***ReaderCons*; **e***SciCons*; **f** pairwise correlations between *AGW* and *ReaderCons* for all four conditions. The consensus items (*ReaderCons* and *SciCons*) use a percentage scale (0–100) and all other items use a five-point scale from “Strongly Disagree” to “Strongly Agree.” See Table 2 for wording of the test items

The five items probing climate attitudes were reverse-scored as appropriate (see Table 2 for a description of all items) and then averaged to form a single composite score, called *AGW* in the figures, that was used for the descriptive statistics. Figure 1 shows summary statistics and distributional information for all dependent variables across the four cells of the experimental design (panels a–e), and pairwise correlations between perceived reader consensus and acceptance of AGW for the four cells separately (panel f). Table 3 shows the cell means for the same set of dependent variables. Skewness and kurtosis for all measures were within ± 1 and there were no particularly outlying observations based on the interquartile range outlier detection rule (Hoaglin & Iglewicz, 1987).

**Table 3:** Means of principal dependent measures across experimental conditions

Type of post	Type of comments	*Unaffected*^*a*^	*ReaderCons*^*a*^	*Support*^*a*^	*AGW*^*a*^	*SciCons*^*a*^
Reject AGW	Endorse AGW	2.97	47.8	3.10	3.19	58.5
	Reject AGW	3.06	63.5	3.46	2.99	52.3
Endorse AGW	Endorse AGW	3.08	64.7	3.44	3.41	66.5
	Reject AGW	2.98	48.1	3.05	3.40	62.1

^a^ See Table 2 for explanation of variable names

The variables in Fig. 1 and Table 3 were analyzed by frequentist as well as Bayesian techniques. We report the Bayesian analysis in the Appendix; here we present a series of 2 × 2 ANOVAs that explored the pattern in Fig. 1. Except where noted, the Bayesian analysis supported identical conclusions. For the item querying whether readers felt they were affected by blog comments (item *Unaffected* in Table 2), no effects reached significance, all *F* < 1. The overall mean of this item also did not differ from the mid-point of the scale (*M* = 3.02, *t*(392) < 1), suggesting that participants were ambivalent about whether or not they were affected by others’ views and this ambivalence was invariant across all conditions.

People’s support for the blog post (item *Support*) was not affected by the type of post, *F*(1,389) < 1, or the type of comments, *F*(1,389) < 1, although there was strong evidence for the interaction of both variables, *F*(1,389) = 10.75,*p* < 0.001, partial *η*^2^ = .027, Cohen’s *F* = .166. This reflected the fact that when the post and comments were of the same type (both rejecting or both endorsing AGW), people supported the post more than when the polarities of the post and the comments were in opposition. This result establishes that reading the comments affected people’s views of the post. Supportive comments enhanced endorsement of the post whereas critical comments undermined that support.

The *AGW* composite score was similarly unaffected by the type of comments, *F*(1,389) = 1.59,*p* > 0.1, although there was very strong evidence of a role of the type of post, *F*(1,389) = 14.28,*p* < 0.0001, partial *η*^2^ = .035, Cohen’s *F* = .192, indicating that people accepted global warming more after reading the post supporting mainstream science than after reading a contrarian post. The interaction between the two variables was non-significant, *F*(1,389) = 1.42,*p* > 0.1. In this instance, however, the subset analysis of careful readers additionally returned a main effect of type of comment, *F*(1,179) = 6.58,*p* < 0.02, reflecting the fact that contrarian comments reduced acceptance of the mainstream science among careful readers.

The perceived consensus among blog readers (item *ReaderCons*) was affected neither by the type of post, *F*(1,389) < 1, nor the type of comments, *F*(1,389) < 1, but it was strongly affected by the interaction of both variables, *F*(1,389) = 50.44,*p* < 0.0001, partial *η*^2^ = .115, Cohen’s *F* = .360. That is, similar to people’s expressed support for the blog post, their perception of consensus among readers was greatest when the comments were consonant with the post (both rejecting or both endorsing AGW) as opposed to when there was a mismatch in polarity. The subset analysis of careful readers additionally returned a main effect of type of post, *F*(1,179) = 5.80,*p* < 0.02, reflecting the fact that more readers were presumed to endorse the science-based post than its contrarian counterpart.

For the presumed consensus among scientists (item *SciCons*), by contrast, what mattered strongly was the type of post, *F*(1,388) = 12.96,*p* < 0.0003, partial *η*^2^ = .032, Cohen’s *F* = .183,^3^ reflecting the fact that the science-based post underscored the scientific consensus whereas the contrarian post undermined it. A weak effect for the type of comments, *F*(1,388) = 4.56,*p* < 0.05, partial *η*^2^ = .012, Cohen’s *F* = .108, was not confirmed by the Bayesian analysis (see Appendix). The interaction between both variables was non-significant, *F*(1,388) < 1.

Taken together, the analyses support two main conclusions: First, the type of post strongly affected people’s attitudes towards climate change, with the science-based post increasing belief in global warming relative to a contrarian post. By contrast, there was no evidence that comments alone affected attitudes towards climate change directly when all participants were considered. Only when we focused on careful readers did a direct effect of comments on climate attitudes emerge, such that comments that endorsed the science were associated with higher acceptance than contrarian comments.

Second, the match or mismatch between post and comments mattered to people’s endorsement of the post and the perceived consensus among readers: critical comments (e.g., contrarian comments following a science-based post or vice versa) undermined support and perceived consensus, whereas favorable comments increased both. In light of the strong effects of comments on perceived consensus among readers, and in light of the direct effect on attitudes among careful readers, we next explored the possibility that reader comments may affect AGW attitudes *indirectly*, via their effect on consensus.

### Inter-variable associations and structural equation modeling

This analysis modeled people’s acceptance of AGW as a function of the two experimental design variables and perceived endorsement among readers. We applied process analysis (Hayes, 2018) to model the relation between those intertwined variables. We first devised a measurement model associated with the five items that queried climate attitudes (items *agw1* though *agw5*). The single-factor model associated with an AGW latent variable was found to be moderately well-fitting, *χ*^2^(5) = 66.64,*p* < 0.001, *CFI* = .910,*TLI* = .821, *RMSEA*= .177, *SRMR*= .069. However, there was an indication that a non-negligible amount of covariance was shared between the two items with negative polarity, *agw1* and *agw5* (see Table 2). With the addition of the covariance between these two items (*r* = .40,*p* < 0.001), the model fit substantially better, *χ*^2^(4) = 6.39,*p* > 0.1, *CFI* = .997, *TLI* = .991, *RMSEA*= .039, *SRMR*= .017. We therefore used this single-factor latent variable as our criterion variable for the remaining modeling.

We next examined some of the key bivariate correlations. The association between perceived scientific consensus (*SciCons*) and the AGW latent variable was large, *r* = .59,95*%CI* : .51,.66, *p* < 0.0001. By contrast, the overall correlation between perceived consensus among readers (*ReaderCons*) and the AGW latent variable escaped significance, *r* = .10,95*%CI* : −.03,.22, *p* > 0.05. The overall absence of a correlation is unsurprising in light of the opposing directions of the relationship between reader consensus and endorsement of AGW across conditions (shown in panel f in Fig. 1). For the posts that rejected science, the correlations between perceived reader consensus and the AGW latent variables were negative/near zero (*r* = −.16 and *r* = .05), whereas they were positive for the posts that endorsed the mainstream scientific position (*r* = .45 and *r* = .25). The reversed directionality between types of post points to a coherent role of the presumed prevalence among readers of support for the scientific position. That is, a negative correlation between the presumed share of readers who endorsed a rejectionist post is equivalent to a positive association between the share of readers who disagreed with that post and, by implication, endorsed the mainstream scientific position. Thus, to simplify presentation of the results, the perceived reader consensus scores provided by participants who were exposed to the rejectionist post were therefore reflected to express *dissensus* with the post (i.e., 100 - *ReaderCons* score). This reflected score represented the perceived endorsement of mainstream science among readers. This reflected measure behaved consistently across conditions was found to correlate moderately with AGW across all conditions, *r* = .25,95*%CI* : .12,.37, *p* < 0.0001.

Finally, we estimated the correlations between the two experimental variables, type of post and type of comments, and the AGW latent variable. For both experimental variables, endorsement of AGW was coded as 1 and rejection as 0. The correlations were *r* = .18,95*%CI* : .07,.27, *p* < 0.003 for type of post, and *r* = .04,95*%CI* : −.08,.15, *p* = 0.520 for type of comments, respectively. Those correlations mirror the ANOVAs reported in the previous section, and they confirm that there was no direct (or total) effect between one of the key experimental variables, type of comments, and AGW. However, contemporary process analysis does not require the presence of a significant total effect, as the observation of an indirect effect by itself is considered sufficient (Hayes, 2009; Preacher & Hayes, 2004; Mathieu & Taylor, 2006; MacKinnon, Krull, & Lockwood, 2000). We therefore explored several candidate process models to explore whether the type of comment may still affect AGW attitudes via an indirect route.

**Fig. 2:**
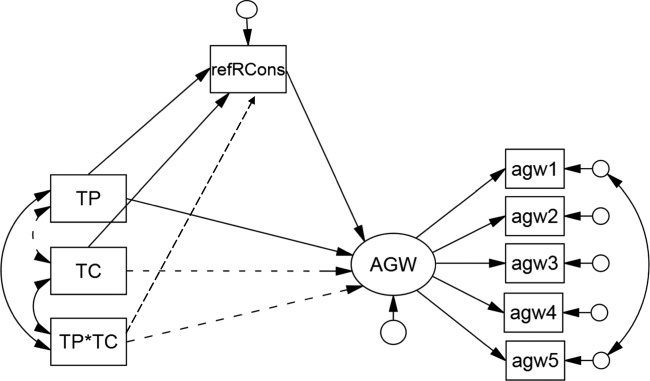
Mediation model with *AGW* regressed upon the experimental variables type of post (TP), type of comments (TC), and a TP × TC interaction term (TP*TC). The reflected perceived reader consensus score (abbreviated to *refRCons*) is the hypothesized mediator. Significant weights and correlations are indicated by *solid lines* and non-significant weights and correlations by *dashed lines*

The first model is shown in Fig. 2. Both experimental variables and their interaction were used to predict AGW directly and indirectly, via the presumed mediator of reflected perceived reader consensus. This model was found to fit acceptably well, *χ*^2^(20) = 51.28,*p* < 0.001, *CFI* = .975, *TLI* = .955, *RMSEA* = .063, *SRMR*= .049. However, several of the estimated effects were not statistically significant (indicated by dashed lines in the figure). In particular, the interaction between the two experimental variables, type of post and type of comments (TP × TC), was not associated with either a direct (*b* = −.16,*β* = −.11,*p* = 0.22) or indirect (*b* = .01,*β* = .01,*p* = 0.79) effect on *AGW*. Furthermore, the type of comments (TC) did not have a direct effect on *AGW* (*b* = .04,*β* = .03,*p* = 0.75).

**Fig. 3:**
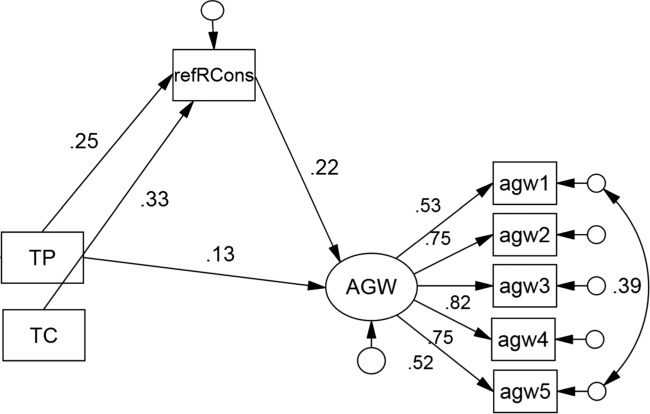
Final process model with completely standardized effects. *AGW* is regressed upon the experimental variables Type of Post (TP) and Type of Comments (TC). The reflected perceived reader consensus score (abbreviated to *refRCons*) serves as mediator. Only significant weights and correlations are shown

Consequently, a revised and simplified process model was estimated which excluded the interaction term between the experimental variables, as well as the direct effect of type of comment on *AGW*. The revised model is shown in Fig. 3 and was found to be associated with acceptable levels of model fit, *χ*^2^(17) = 45.93,*p* < 0.001, *CFI* = .964, *TLI* = .941, *RMSEA*= .066, *SRMR*= .052. All weights and correlations shown in the figure are significant. It can be seen that the type of post (TP) had a direct effect on *AGW* (*b* = .16,*β* = .13,*p* = 0.007). Thus, exposure to the scientific post was associated with greater levels of acceptance of the mainstream science, as already indicated by the corresponding main effect in the earlier ANOVA. A corresponding direct effect for type of comments (TC) was absent. However, both types of post, *b* = .07,*β* = .05,*p* = 0.002, and type of comment, *b* = .09,*β* = .07,*p* = 0.001, had indirect effects on *AGW* via the reflected reader consensus score. Thus, people’s acceptance of mainstream science was in part determined by their perception of how many other readers shared their view, and that perception in turn was influenced by our experimental design variables, namely the type of post and type of comments. The multiple *R* associated with the model was .280 (*p* = 0.004). Thus, 7.7% of the *AGW* true score variance was accounted for by the direct effect of the type of comment variable and the indirect effects of both experimental variables.

## Discussion

Our results are readily summarized: (a) We observed symmetrical effects of blog comments on readers’ endorsement of the post, such that whenever the comments agreed with the post, participants supported the argument in the post more, irrespective of its content. (b) The same pattern was obtained for the perceived consensus among readers. (c) Concerning attitudes towards AGW, comments had a direct effect only with careful readers but not the full sample. (d) The extent to which comments provided information about a consensual endorsement of AGW among other readers indirectly determined participants’ attitudes towards AGW. Reports of indirect effects in the absence of total effects are not uncommon; see, e.g., Kohen, Leventhal, Dahinten, & McIntosh, 2008, Waldzus, Mummendey, Wenzel, & Weber, 2003.

Although our indirect effect was small in absolute magnitude, it may be of some practical significance when it is scaled up to the number of readers of scientific blogs (for other examples of the relevance of seemingly small effects, see Kahan & Braman, 2003). Specifically, the observed difference in perceived reader consensus between the two comment conditions involving the mainstream scientific post can be converted into the expected change in AGW acceptance based on the model in Fig. 3. When this is done, contrarian comments are associated with a decline in acceptance of AGW by .13 on the items’ five-point scale.^4^ Given that 13.5% of our sample scored 3 on the AGW items, representing exact neutrality or complete indifference, the contrarian comments may suffice to nudge those respondents off the fence and towards rejection (i.e., a score below 3). Scaling up this effect to an audience of 1.5 million of popular blogs (Batts et al., 2008), our results suggest that a large number of readers may be nudged towards rejection of climate science if they encounter a stream consisting of contrarian comments.

The potential societal significance of comments appears particularly disproportionate in light of the minute fraction of readers who write comments. To illustrate, only .06% of users of National Public Radio (NPR) in the U.S. were estimated to leave comments (Jensen, 2016).

The fact that a small fraction of readers who leave comments—some of whom may even be “sock puppets” (Bu et al., 2013; Lewandowsky, 2011)—can leverage public opinion about scientific issues must be of concern. This concern appears widely shared among Internet news services, although there is disagreement about the appropriate response. One response has been to discontinue commenting facilities altogether. Several large online news services have taken this step, including NPR (Jensen, 2016), *Popular Science* (http://www.popsci.com/), and *Vice* news (https://www.vice.com). Users can now also download a browser add-on that automatically shuts out comments unless explicitly enabled by the user (https://rickyromero.com/shutup/). Other alternatives include strict moderation of comments, as for example trialed by the online newspaper *TheConversation.com.* which has entertained options such as a “community council” to provide moderation (https://theconversation.com/involving-a-community-council-in-moderation-25547). A further alternative that is being explored by a Norwegian site is the requirement that readers must pass a brief comprehension quiz before being permitted to post comments (http://www.niemanlab.org/2017/03/this-site-is-taking-the-edge-off-rant-mode-by-making-readers-pass-a-quiz-before-commenting/).

Turning to theoretical implications, we have framed our study and results in terms of the importance of the perceived consensus on attitudes towards scientific issues. There is growing evidence that knowledge of the overwhelming scientific consensus on climate change can shift people’s AGW attitudes (Cook & Lewandowsky, 2016; Harris, Sildmäe, Speekenbrink, & Hahn, 2018; Lewandowsky et al., 2013; van der Linden et al., 2015; van der Linden, Leiserowitz, & Maibach, 2018). Likewise, knowledge of the consensus among medical scientists about the efficacy of vaccinations has been shown to increase public support for vaccines, and concomitantly reduce concern about their safety (van der Linden et al., 2015). Our results are fully consonant with those findings but additionally extend the importance of perceived consensus to other sources, in this case anonymous other readers of blogs. Our result meshes well with a recent study by Harris et al., (2018), who examined the efficacy of various consensus-based messages on shifting people’s attitudes towards climate change. One of their studies used an American online sample and is thus comparable to the present experiment. Harris et al., found that the most effective way to communicate the scientific consensus was by “experiencing” it—that is, by being exposed to individual opinions from a number of scientists—as opposed to being given a summary statistic (e.g., “97 out of 100 scientists agree on AGW”). Their results confirm the power of experiential exposure to opinions; we extend those results by showing that even anonymous voices with unknown expertise can shape people’s experience of an opinion-relevant consensus.

There are, however, other theoretical frameworks that could be applied to the present results. For example, Walther, Woo Jang, and Edwards (2018) examined the effects of user-generated online health advice within the heuristic–systematic model of social influence (Chaiken, 1980) and warranting theory (Walther, Heide, Hamel, & Shulman, 2009). Those models present a more nuanced and complex route to social influence; however, both subsume heuristics such as the effects of consensus explored here. Thus, within the heuristic-systematic model, a perceived consensus might trigger attitude change based on a “consensus implies correctness” heuristic (Todorov, Chaiken, & Henderson, 2002). Within warrant theory, the presence of multiple seemingly independent sources can likewise provide epistemic warrant for the shared underlying opinion that is being articulated (Walther et al., 2018). Our results do not differentiate between those nuanced theoretical positions.

We conclude by highlighting several open questions for researchers that our results helped bring into focus. First, how much dissent in a comment stream is required to disrupt the perception of a widespread consensus? Our streams were uniform in their orientation towards one position or the other, but in reality this rarely occurs. Might a single deviating comment be sufficient to disrupt consensus perception, as is suggested by the results of Koehler (2016) in a simulated “journalistic-balance” environment? Or might a pervasive consensus be perceived even when the share of supporting comments is at only 70%, as is suggested by some quantitative models of social influence (Muthukrishna et al., 2016; Muthukrishna and Henrich, 2019)? Second, can the effects of comments be moderated by information about hidden base rates (e.g., how many readers did the post have, relative to the number of comments; what was the proportion of “likes” and “dislikes” among readers who did not comment?). Third, to what extent do the self-report measures explored here translate into actual political behaviors, such as increased climate activism or disengagement with the issue. In light of current concerns about the erosion of truthful public and political discourse (Lewandowsky, Ecker, & Cook, 2017; Lewandowsky, Cook, & Ecker, 2017), those questions await urgent exploration.

## Appendix A: Bayesian analysis

We used the BayesFactor package for R (Morey, 2015) with the “bottom” option, which adds single effects one at a time in comparison to the null model. The BayesFactor package calculates the Bayes factor (BF) for pairwise model comparisons, using default priors on the effect sizes included in a model (Rouder, Morey, Speckman, & Province, 2012). The BF reflects the relative strength of evidence for one model over the other that is provided by the data, independent of the researcher’s prior beliefs. Bayes factors quantify the strength of evidence on a continuous scale, and unlike with *p* values, there is no conventional cut-off. A BF equal to 1 reflects complete ambiguity in which the evidence favors both models equally; BFs between 1 and 3 are considered weak evidence, BFs between 3 and 10 as intermediate, BF > 10 as strong evidence, and BF > 100 as decisive evidence in favor of one model against the other (Kass & Raftery, 1995). When the BF falls below one, its reciprocal can be interpreted in the same manner but with reverse polarity—that is, a Bayes Factor of 0.1 for a given model is actually strong evidence (1/0.1 = 10) *against* it (and in favor of the other model).

The Bayesian analyses on the full sample of participants largely paralleled the results of the frequentist analysis. For the *Unaffected* item, there was no evidence for the inclusion of either main effect or the interaction compared to a null model with the grand mean only; largest BF = 0.15. People’s support for the blog post (item *Support*) was not affected by the type of post (BF = 0.11) or the type of comments (BF = 0.11), although there was strong evidence for the interaction of both variables (BF = 19.04). The *AGW* composite score was unaffected by the type of comments (BF = 0.21) or the interaction (BF = 0.19), although there was very strong evidence of a role of the type of post (BF = 80.74).

For the two consensus items, the perceived consensus among blog readers was affected neither by the type of post (BF = 0.12) nor the type of comments (BF = 0.12), but it was decisively affected by the interaction of both variables (BF = 1.50 × 10^8^). For the presumed consensus among scientists (*SciCons*), by contrast, what mattered strongly was the type of post (BF = 42.84) but not the comments (BF = 0.11) or the interaction (BF = 0.79).

## Footnotes

1 We use the term “blog” as a short-hand for a variety of social media sites, including for example YouTube, that permit user-generated content and in particular public comments. Those sites that permit generation of user content are collectively also known as Web 2.0 (Kata, 2012).

2 Age was self-reported using a slider with end points 15 and 100. The minimum reported age was 18 and the maximum 100, with eight participants reporting an age above 90. Because all participants passed the a priori comprehension criterion, these observations were retained for analysis.

3 This variable had one missing observation, which explains why the denominator *df* are different from all the others for this test.

4 The difference in perceived consensus between the two types of comments is 16.64, which translates into .74 of a standard deviation. The correlation between *refReadCons* and AGW was *r* = .19, implying that a 1 SD decrease in perceived consensus corresponded to a .19 SD decrease in AGW. Given that the standard deviation of AGW across all participants was .87, the expected shift downward in AGW acceptance as a function of contrarian comments was .74 × .19 × .87, or .13 on the 5-point scale.
